# Chondral delamination of the knee and its management: a case report and review article

**DOI:** 10.1186/s12893-022-01775-w

**Published:** 2022-08-29

**Authors:** Marah Mansour, Yazan Abboud, Mhd Alaa Aldin Alhaffar, Ola Souliman, Massa Haffar, Younes Mustafa, Tamim Alsuliman, Michel Samaan

**Affiliations:** 1 Faculty of Medicine, Tartous University, Tartous, Syrian Arab Republic; 2 Karsh Division of Gastroenterology and Hepatology, Cedars-Sinai Medical Center, Los Angeles, CA USA; 3 Respiratory Department, St Mary’s Hospital, Imperial College Healthcare NHS Trust, London, UK; 4 Faculty of Medicine, Syrian Private University, Damascus, Syrian Arab Republic; 5 Faculty of Medicine, Alexandria University, Alexandria, Egypt; 6 Hematology and Cell Therapy Department, Saint-Antoine Hospital, AP-HP, Sorbonne University, Paris, France; 7 General Secretary of the Syrian Association of Arthroscopy SSA, Department of Orthopedic Surgery, Faculty of Medicine, Al-Baath University, Homs, Syrian Arab Republic

**Keywords:** Chondral delamination, Knee lesions, Microfracture, Degenerative arthritis, Cartilage, Arthroscopy

## Abstract

Chondral delamination is the separation or debonding of the articular cartilage from the underlying subchondral bone. The hyaline cartilage has a limited capacity for healing, meaning it does not possess the innate ability to restore its normal structure or to heal the subchondral bone once detached from it. The purpose of this article is to report the outcomes of a microfracture technique used to manage chondral delamination in a 59-year-old male; we also reviewed the treatment options mentioned in the literature. The patient was admitted to the Department of Orthopedic Surgery complaining of recurrent severe right knee pain with multiple episodes of knee locking, denying any direct or twisting trauma to the knee. The plain X-ray showed mild degenerative changes with articular surface irregularity. On Magnetic resonance imaging, wide chondral delamination was noted in the medial femoral condyle. After 12 months’ post-op, his condition improved. No locking was observed. Pain improved in comparison to the pre-operative levels. The international knee documentation committee improved from 26.4% to 52.9%. In a follow-up magnetic resonance imaging, the adhesion of most parts of the delaminated cartilage.

## Introduction

Hyaline cartilage is the cornerstone in the anatomy of almost all joints in the body, especially synovial ones. It is an avascular, aneural, and alymphatic tissue that plays a major role in their movement. Chondral delamination (CD) is the separation or debonding of the articular cartilage from the underlying subchondral bone at the tidemark forming an unstable cartilage flap that is at risk for complete detachment from the adjacent cartilage, causing full-thickness defects and intra-articular loose bodies [1–5]. Structurally, Hyaline cartilage is composed of chondrocytes, an extracellular matrix of water, type II collagen, and proteoglycans. The interplay between the solid and fluid components gives the cartilage its biphasic and viscoelastic properties that are crucial not only for its shock-absorbing ability but also to provide an almost frictionless articulation [4]. The structure of an articular hyaline cartilage can be said to contain two large zones, a calcified and a non-calcified zone. The non-calcified zone may be further subdivided into the superficial zone, in which collagen fibers are arranged parallel to the surface and offer good resistance to shear force. Transitional zone, in which collagen fibers run obliquely, and deep zone, where collagen fibers are oriented perpendicularly to the surface, thus resistant to compression. The calcified zone of cartilage contains cartilage fibers, anchored by hydroxyapatite crystals, to the subchondral bone. The junction between the calcified and noncalcified zone is the tidemark [4, 6]. The hyaline cartilage has a limited capacity for healing meaning it does not possess the innate ability to restore its normal structure or to heal the subchondral bone once detached from it, so its preservation is paramount for joint health and mobility [4, 7–9]. Delamination may occur with or without degenerative changes. It usually occurs in adults in their 3rd and 4th decade. Adolescents do not develop this lesion, because the tidemark is not well-developed until approximately the age of 20. Alternatively, osteochondral fractures can occur [10]. Clinical experiences have demonstrated the frequency of chondral lesions in athletes [11]. Although acetabular CD can be found in many joints, it is a frequent finding in hip arthroscopy [12]. Eugeno Jannelli et al. described the histological changes in the delaminated area(s). They noted the presence of hypocellularity associated with fragmentation and fissuring of the matrix with no bone involvement. These changes extend to reach the subchondral bone but do not involve the articular surface. The matrix showed a considerable architectural disorder with diffuse eosinophils and myxoid degeneration foci. The collagen fibrous layer appears inverted in structure and orientation. Chondrocytes presented in a nonhomogeneous distribution and were relatively more numerous in the deepest chondral layer. We also occasionally observed superficial microfoci with a slight increase in cellularity. Delamination can occur in all three compartments of the knee, especially on the femoral side [3]. The delamination line runs parallel to the joint surface. During arthroscopy, if there is no disruption of the cartilage surface it appears as what is known as the “carpet phenomenon”; which means the cartilage layer can move relative to the underlying bone plate, similar to a carpet on a slippery floor. Although the cartilage may stay intact, it loses its function as an anchor, which leads to higher odds of further damage either by the spread of the delamination or the invasion of the articular surface with the subsequent formation of chondral flaps [11]. However, if the cartilage is disrupted, the detached cartilage flap then becomes visible. Thus, it will be possible to introduce a probe between the deboned cartilage and the subchondral bone [1, 3, 5].

## Background and purpose

Chondral delamination is the separation “deboning” of the articular cartilage from the subchondral bone that lies underneath. The hyaline cartilage has little ability to heal, and it does not possess the innate ability to restore its normal structure or to heal the subchondral bone once detached from it. The purpose of this report was to show the early results of chondral delamination treatment in a 59-year-old man using the microfracture technique and to a review the treatment modalities in managing these lesions from the literature.

## Case presentation

**Fig. 1:**
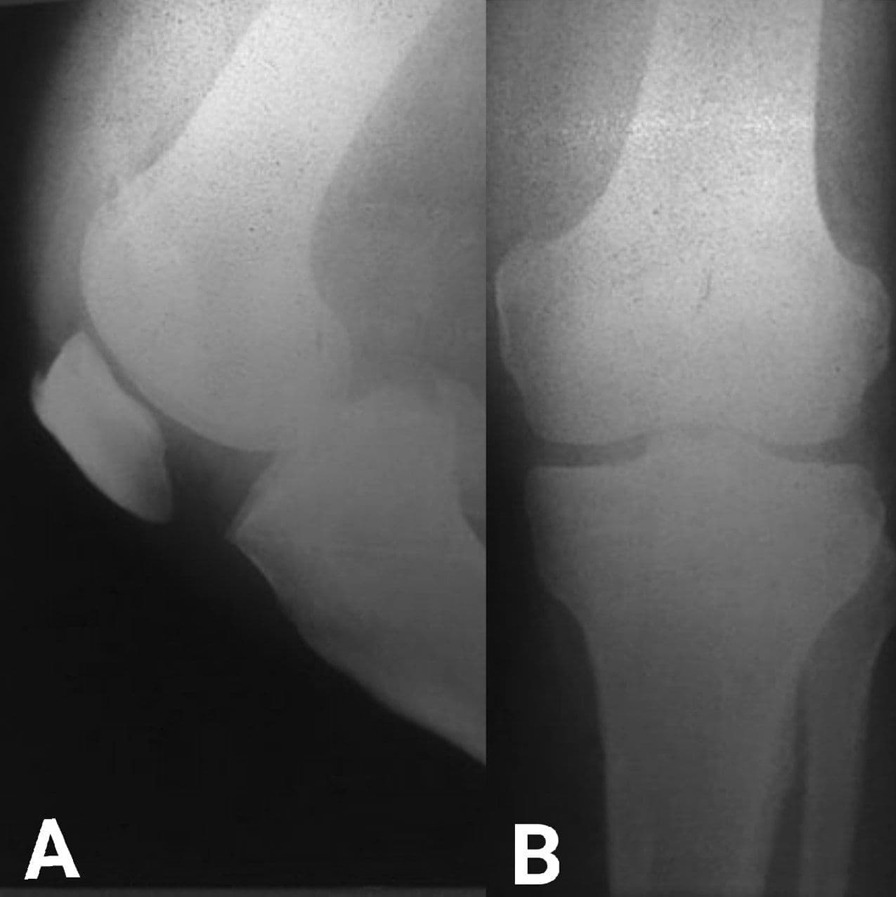
**A**, **B**: Pre-operation plain X-ray shows mild degenerative changes

**Fig. 2:**
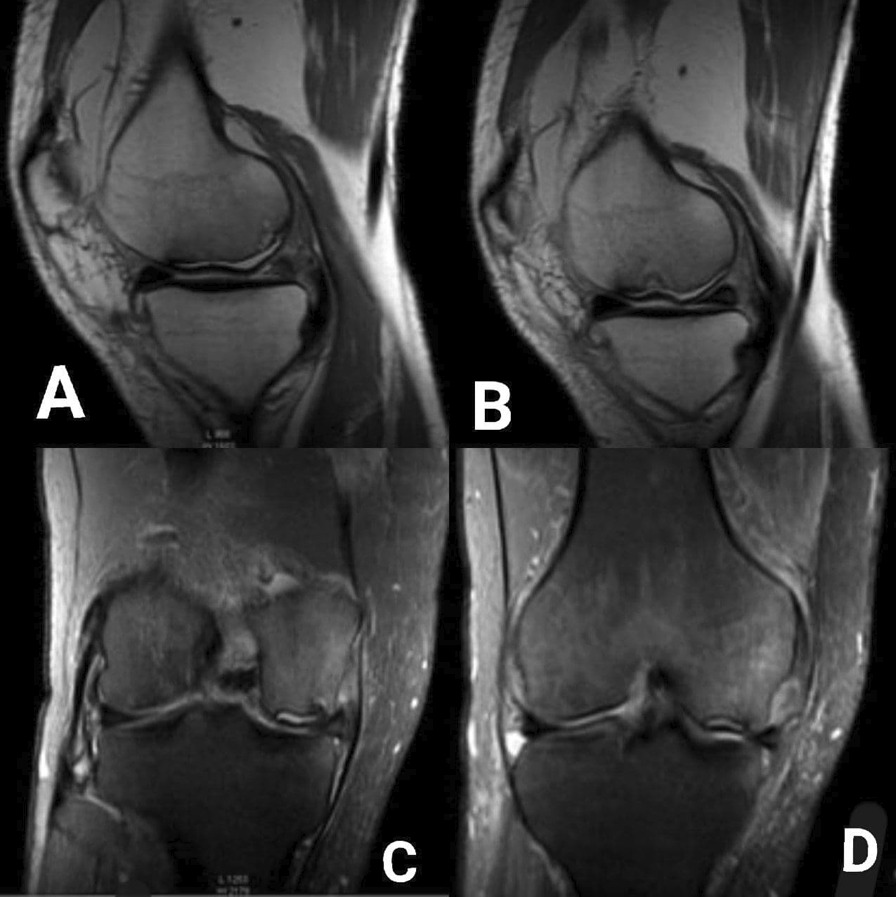
**A**–**D**: **A** PDW-aTSE sequence sagittal image showing the delaminated flap with subchondral bone sclerosis and irregularity. **B** PDW-SPIR sequence coronal image showing the delaminated flap with subchondral bone sclerosis and irregularity and medial condyle edema

**Fig. 3:**
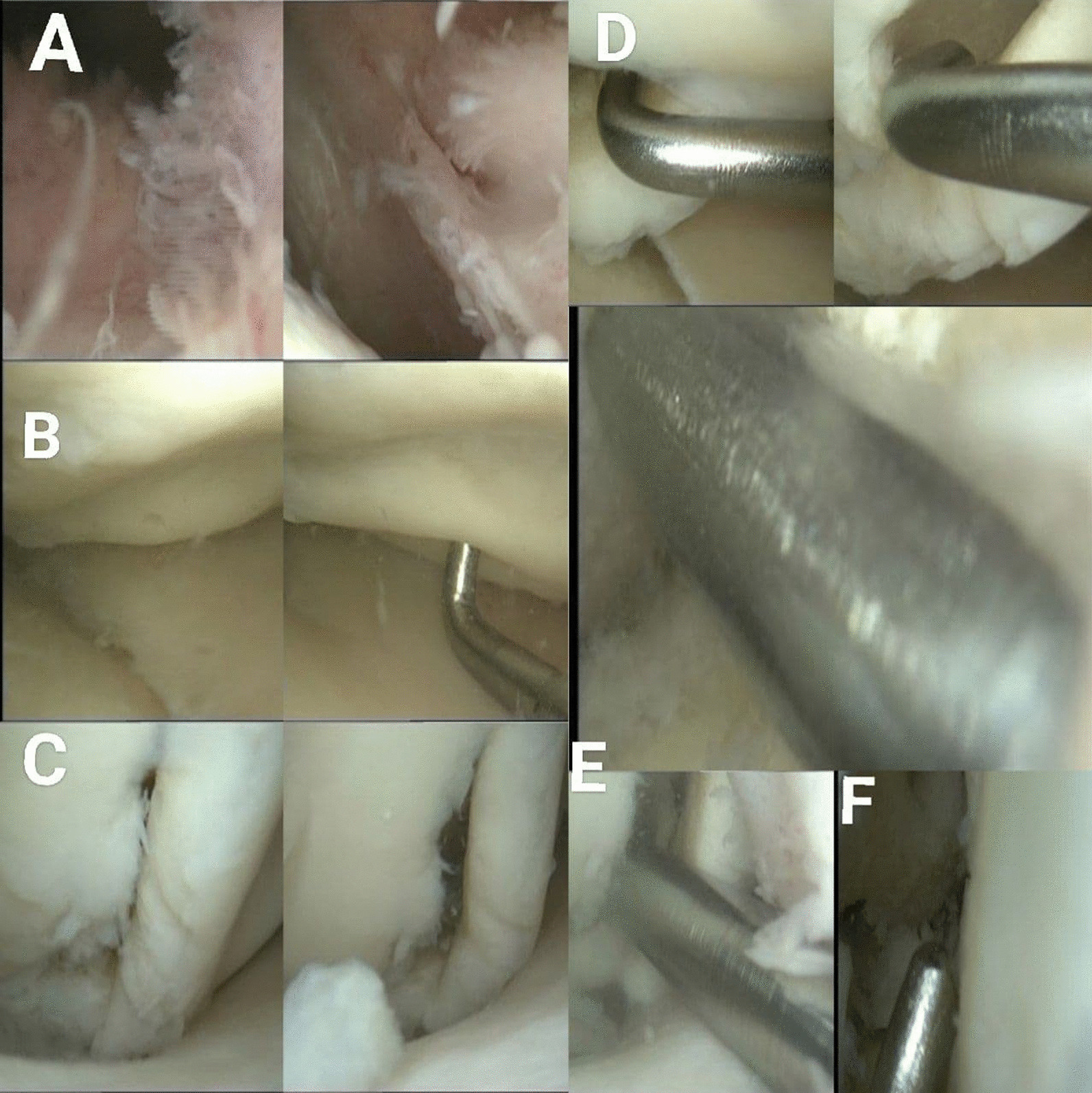
**A**–**F**: **A** Diffused synovitis was seen during the arthroscopy. **B** Carpet phenomenon. **C** Ruptured cartilage flap. **D** The passage of the arthroscopic hook between the flap and the subchondral bone. **E** Using a drill bit and a wire of 1.2 mm multiple passages have been done. **F** The final appearance of the holes above the cartilage flap

**Fig. 4:**
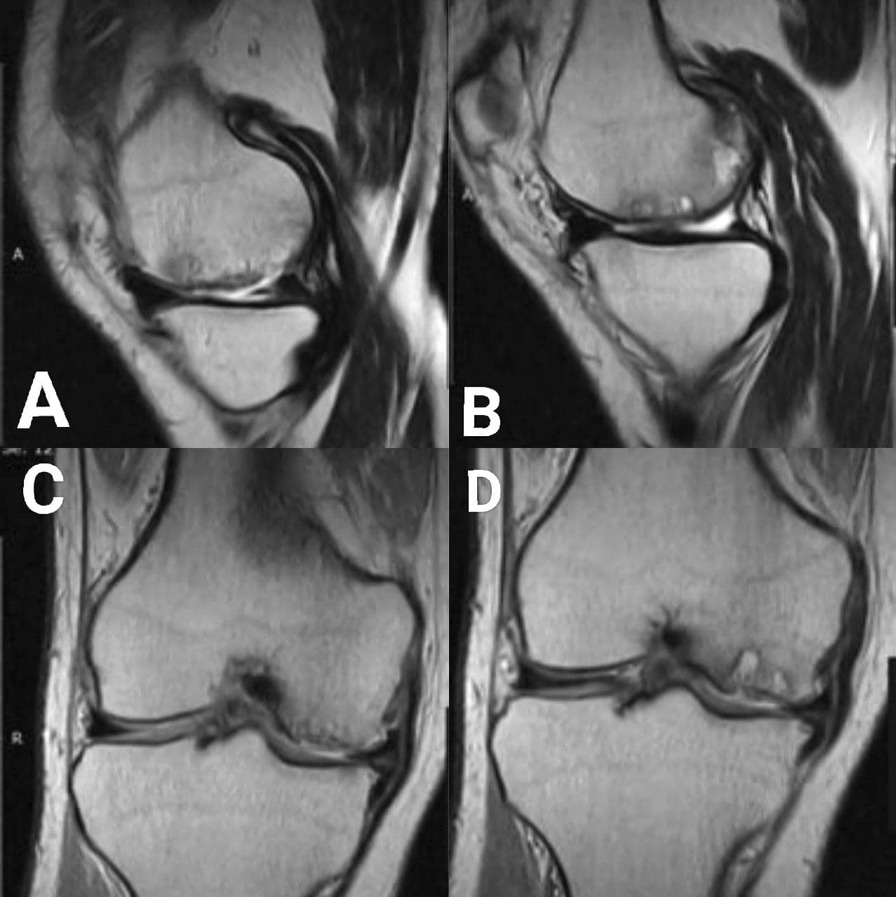
**A**–**D**: **A**, **B** T2 sequence sagittal image showing the adherence of the delaminated flap in most parts (MRI center did not provide sagittal images in PDW sequence). **C**, **D** PDW sequence coronal image showing the irregular subchondral bone and the adherence of the many parts of the delaminated flap with loss of some chondral parts

A 59-year-old male was admitted to the Department of Orthopedic Surgery complaining of recurrent extreme right knee pain after prolonged standing and multiple episodes of knee locking with extreme limitation of its movement, a tender medial knee, and a mild degree of genu varum and mild effusion. A medical history of diabetes mellitus, hypertension, and hypercholesterolemia was recorded, but no history of direct or twisting trauma to the knee was observed. The patient was not sportive but used to climb stairs quickly with 165 cm in height and 75 kg in weight, resulting in a BMI of 27.5. On clinical examination, the right knee range of motion (ROM) was 0\0\100° with clear crepitus during motion. The IKDC (International Knee Documentation Committee) was 26.4%. The plain X-ray showed only mild degenerative changes that fit under grade 1 which in turn is the possible narrowing of the joint space with the probable formation of osteophyte according to Kellgren-Lawrence Classification of Osteoarthritis Fig. 1A, B. Magnetic resonance imaging (MRI) showed wide chondral delamination in the medial femoral condyle Fig. 2A–D. During arthroscopy, obvious synovitis was noted, and a large ruptured chondral flap was seen (measuring about 2 × 3 cm) carpet signs or wave phenomenon was obviously noted with exposure of the subchondral bone through the ruptured flap Fig. 3A–F. Because of the advanced age of the patient, the subchondral bone irregularity, and the beginning of arthritic changes in the knee, the Microfracture technique (the simplest and cheapest technique), were applied using a drill-bit and a wire of 1.2 mm to perforate the subchondral bone. Curettage of the calcific layer by an arthroscopic curette was also used. No debridement was done to the flap. In the postoperative period, the rehabilitation protocol consisted of a non-weight bearing period of 45 days without limitation of motion, followed by a progressive restoration of the range of motion of the knee and muscle strengthening by closed chain exercises. Full weight bearing and independent motion were restored 3 months after the operation. The goal was to try to adhere this flap to the subchondral bone by creating a bleeding surface to slow the progression of degenerative arthritis that already has begun, and to relieve the mechanical derangement caused by the mobile flap. During the postoperative period, the non-weight bearing was continued until 6 weeks, with a gradual return to activity. Analgesics and glucosamine supplements were used as well. After 12 months’ post-operation, the general complaint of the knee has improved. No locking was observed. The pain improved from 7\10 in pre-operation to 3\10 in post-operation (considering 0 = no pain, and 10 = intolerable pain) in comparison to the pre-operative levels. The IKDC became 52.9%. The patient satisfaction was very good. A follow-up MRI showed the adhesion of most parts of the delaminated cartilage Fig. 4A–D. Although knee degeneration did not improve, the general activity of the knee remarkably improved, and the general symptoms were ameliorated. The reason for this amelioration was potentially the preservation of the cartilage of good thickness and the stability of most parts of the chondral flap.

## Discussion and conclusion

CD is a lesion that may jeopardize the future of the joint if misdiagnosed. Cartilage lesions were classified by many authors and societies. The most used classification is (Outbridge 1961) [1]. Many other classifications were proposed such as the ones proposed by F.R. Noyes et al [2] and ICRS [3] but chondral delamination was clearly included in Konan et al.’s classification (where grade 0 represented normal articular cartilage lesions, grade 1 represents softening or a wave sign, grade 2 cleavage lesion, grade 3 delamination and grade 4 bone exposure) [4]. The cause of cartilage delamination is believed to occur through different mechanisms: large shear force concentrated at the junction of the cartilage and the calcified layer disrupting the deep cartilage ultrastructure. This force can produce damage to the cartilage above the tidemark and to the subchondral bone [3, 10, 11]. Less frequently, the direct blow can be presented as a possible cause [10]. Additionally, abrasive wear or friction may result in fibrillation and subsequently delamination [11]. Although mechanical trauma is associated with some types of chondral injury. Many patients with CD are unable to relate any history of trauma, attributing all such injuries to acute causes is an oversimplification. Yet unknown structural and biomechanical changes may still significantly contribute to the production of chondral delamination [10]. One example of these mechanisms is Synovial plicae that may cause injuries to the underlying cartilage through a combination of compression, friction, and shear forces. Furthermore, the increasing young’s modulus of the stiff medial parapatellar plica is associated with greater contact pressures on the underlying cartilage [6]. Another example is unstable knees (as in ACL deficient knees) that are more prone to initiate the delamination which may progress to cause locking of the chondral flap [11]. The complaint of patients is usually aching or sharp pain during and after activity. This pain may be diffused or localized. The size and the location of the lesion strongly affect the type of symptoms and may affect surgical treatment options [2, 10]. For example, patellofemoral flaps cause anterior knee pain without instability, while posterior condylar flaps cause instability, sometimes they may resemble a torn discoid lateral meniscus or even medial meniscus tear [6]. Swelling and effusion related to the activity are also well mentioned. Joint line tenderness is present, crepitus, thigh atrophy, decreased ROM and Mechanical symptoms such as locking and catching and giving way are also frequent [6, 11]. Early diagnosis of delamination appears to be challenging and important because cartilage detachment is irreversible and has an important effect on the treatment plan [1]. Diagnosis depends firstly on clinical suspicions like the presence of discomfort during or after physical activity associated with effusion and swelling. Sometimes, an accurate diagnosis may be challenging, because it may resemble other pathologies such as meniscal lesions [6]. Plain x-rays are usually normal. The MRI study is fundamental in the evaluation, and mandatory to confirm the diagnosis and to aid preoperative planning [3]. In general, its sensitivity for the detection of articular cartilage injury is significantly lower than that for meniscal injury [6]. Articular cartilage has intermediate to high signal intensity on both T1 and T2 weighted images [4, 10]. Bodelle et al. showed that in the patellofemoral joint, the STIR-sequence is significantly superior to the MEDIC-sequence regarding the depiction of chondral lesions [13]. Delamination may require fluid beneath the disrupted cartilage to be evident on the MRI scan [11]. Although MRI helps detect the site and the size of the lesion, it is sometimes difficult to diagnose these lesions because of several factors: lack of awareness of the diagnosis on the part of the radiologists, inappropriate MRI techniques, or inherent limitations of MRI to detect this lesion [3]. The negative MRI scan does not rule out delamination lesions [10, 11]. The clinician must rely not only on MRI scan evaluation but also on the complete clinical picture [10]. Arthroscopy remains the best method to confirm chondral injury diagnosis [10]. It should be performed to confirm the diagnosis with visualization and application of the probe to the articular surfaces, to exclude confounding pathology, and to perform a chondroplasty if appropriate [3, 4]. First-line treatment is usually conservative, using analgesics and non-steroidal anti-inflammatory drugs with low-intensity low impact endurance exercises such as cycling and swimming [10]. Surgical treatment options vary according to the site, size, and status of the cartilage continuity. The treatment varies widely from simple debridement of the delaminated flaps to stable margins, and\or removal of loose bodies that improves mechanical symptoms and prevents irritation of the synovium due to small fragments of cartilage being released. Combined with debridement of the calcified cartilage to bleeding bone by curettes or rotary shaver [10]. Another option, if the cartilage surface is intact, is to fix the area of delamination with bioabsorbable pins. This may be done arthroscopically or via a small arthrotomy, depending on the location of the lesion. If lesion size allows, the additional passage of a fine drill through the affected articular surface affected into the related subchondral bone, simulates lesion healing [4]. Some authors recommend the use of fibrin adhesive as a biological substance that has hemostatic and adhesive properties. This fibrin permits tissue fixation and stimulates the growth of fibroblasts [14]. Kaya et al. reported excellent early clinical results for arthroscopic repair of carpet delamination using fibrin glue augmented with bridging suture technique to repair acetabular cartilage carpet delamination [12]. Microfracture is a well-described and extensively studied the technique in which penetration of subchondral bone and subsequent release of the underlying marrow elements lead to the formation of reparative cartilage [1, 7, 8, 11, 14]. It is a commonly performed procedure for defects smaller than 2 cm [12] because, in the case of a large defect, the fibrocartilage patch becomes more unstable and more liable to detach because of clot retraction [8]. Although fibrocartilage tissue is inferior to hyaline cartilage and degenerates with time, it can provide some benefit to the patient. However, it does not retard the progression to OA [4, 10]. Other options are autologous chondrocytes implantation, debridement and the use of a periosteal flap to cover the defect, and mosaicplasty [4, 10]. Osteochondral allografts (OCAs) are another reliable technique for the treatment of large chondral or osteochondral defects. The use of matched OCAs eliminates donor site morbidity, provides immediate structural restoration of the articular surface and allows for the treatment of large lesions [15]. Treatment results and options depend on the location; the size and the age of the patient [1], (Table 1). In this case, curettage and subchondral bone drilling improved the symptoms to an acceptable level and resulted in a satisfied patient. In conclusion, unrecognized and/or untreated chondral delamination injuries have a poor prognosis. Treatment of these lesions, even if they are large, will help improve symptoms and joint mobility. The simplest and most cost-effective method we used seems to be very effective with satisfactory mid-term outcomes. Future studies are needed to further evaluate the microfracture technique in managing chondral delamination.

**Table 1:** Chondral delamination characteristics from the literature

Author, year	Age	Main complain(s)	Treatment	Follow-up
Unverferth, 1998 [10]	NR	NR	NR	NR
Kendell, 2005 [3]	Ranging from 16 to 37	Acute pain in the knee	Surgery	NR
Pfirrmann, 2008 [1]	Ranging from 16 to 49	NR	NR	NR
Anderson, 2009 [2]	Ranging from 16 to 51	Femoroacetabular impingement of the hips in 60 patients, perthes disease in 2 patients, multiple hereditary exostoses in 1 patient, and slipped capital femoral epiphysis in 1 patient	Surgery	NR
Tzaveas, 2010 [14]	Ranging from 18 to 57	Persistent hip pain for a mean period of 19 months	Surgery	Improvement in pain and function six months and one year after surgery without any complains
Bardos, 2015 [9]	Ranging from 14 to 44	Defects in the MFC lateral extension or central extension on weight-bearing surface and a lesion in the patella	Surgery	
Kaya, 2015 [12]	NR	NR	Surgery	Full therapy was advanced when the patient was fully weight bearing and achieving a full range of motion
Jonathan, 2016 [6]	39	Chronic pain in the knee for 3 years	Arthroscopy	Normal activities were resumed 2 weeks after surgery. The knee pain resolved after 7 months
Bodelle, 2016 [13]	Mean of 44 (± 12)	Acute pain at the anterior aspect of the knee, joint effusion and a suspected chondral lesion defect in the patellofemoral joint	NR	NR
Tahoun, 2017 [8]	Ranging from 18 to 50	NR	Hip arthroscopic surgery	The mean HOS for daily live activities and the sports subscale improved. All patients had > 90% of the filling of the chondral defect
Lands, 2017 [5]	57	Pain in the left hip	Surgery	Good alignment of the presence of an uncemented left total hip was found on post-surgical radiographs
Bogunovic, 2019 [7]	NR	NR	Defects were made in the trochlea and MFC of 6 cadaver specimens with application of an allograft	Fibrin delamination and/or allograft displacement occurred within the first 15 min of examination in 82% of the specimens
Theodorides, 2019 [4]	Mean of 26.6 (± 12.8)	Chronic pain in the knee	Arthroscopy, surgery and SmartNails were inserted	All patients had full recovery with improvement in symptoms

*MFC* Medial femoral condyle, *HOS* Hip outcome score

## Data Availability

Not applicable. All data (of the patient) generated during this study are included in this published article and its Additional files.
